# Spatiotemporal walking performance in different settings: effects of walking speed and sex

**DOI:** 10.3389/fspor.2024.1277587

**Published:** 2024-03-15

**Authors:** Jackson Lordall, Alison R. Oates, Joel L. Lanovaz

**Affiliations:** College of Kinesiology, University of Saskatchewan, Saskatoon, SK, Canada

**Keywords:** walking environment, inertial sensors, gait analysis, wearable technology, overground, treadmill, young adults

## Abstract

**Background:**

Understanding the factors that influence walking is important as quantitative walking assessments have potential to inform health risk assessments. Wearable technology innovation has enabled quantitative walking assessments to be conducted in different settings. Understanding how different settings influence quantitative walking performance is required to better utilize the health-related potential of quantitative walking assessments.

**Research question:**

How does spatiotemporal walking performance differ during walking in different settings at different speeds for young adults?

**Methods:**

Forty-two young adults [21 male (23 ± 4 years), 21 female (24 ± 5 years)] walked in two laboratory settings (overground, treadmill) and three non-laboratory settings (hallway, indoor open, outdoor pathway) at three self-selected speeds (slow, preferred, fast) following verbal instructions. Six walking trials of each condition (10 m in laboratory overground, 20 m in other settings) were completed. Participants wore 17 inertial sensors (Xsens Awinda, Movella, Henderson, NV) and spatiotemporal parameters were computed from sensor-derived kinematics. Setting × speed × sex repeated measures analysis of variance were used for statistical analysis.

**Results:**

Regardless of the speed condition, participants walked faster overground when compared to while on the treadmill and walked faster in the indoor open and outdoor pathway settings when compared to the laboratory overground setting. At slow speeds, participants also walked faster in the hallway when compared to the laboratory overground setting. Females had greater cadence when compared to males, independent of settings and speed conditions.

**Significance:**

Particularly at slow speeds, spatiotemporal walking performance was different between the settings, suggesting that setting characteristics such as walkway boundary definition may significantly influence spatiotemporal walking performance.

## 1 Introduction

Quantitative walking assessments can provide valuable information regarding health status. For example, walking speed alone can give insight into an individual's physical independence, fall risk, current and future health status, and responsiveness to rehabilitation efforts (1). Identifying factors that influence walking performance is important to inform the design of walking assessment protocols.

Quantitative walking assessments are often limited to laboratory settings which usually consist of a short walkway or treadmill for data collection due to physical constraints of data collection equipment (e.g., optical motion capture systems). Developments in wearable sensor technology have enabled quantitative walking assessment outside of laboratory settings (2). Consequently, there is evidence to suggest that spatiotemporal walking performance can differ between overground and treadmill laboratory walking (3–8), laboratory and non-laboratory settings (e.g., outdoors) (8–10), and indoor and outdoor non-laboratory settings (8, 11–17). Research investigating the influence of settings on spatiotemporal walking parameters is difficult to synthesize due to differences in instrumentation [e.g., number, type, and location of sensor(s)], protocol (e.g., instructions, walking distances), and sample demographics (e.g., age, health status). These limitations highlight the need for a comprehensive assessment of spatiotemporal walking parameters between multiple settings in the same study to better understand the influence of settings on walking.

Walking at above and below preferred speeds can influence motor control requirements (18, 19); thus, evaluating walking at different speeds may provide insight into the influences of different settings on spatiotemporal walking performance. Increased walking speed results from larger stride lengths (SL) and increased cadence (19, 20), and increased walking speed leads to less time spent in double support (DS) (19).

Sex effects are important to consider when characterizing the factors that may influence spatiotemporal walking performance. A descriptive meta-analysis (21) suggested that, after accounting for body size (e.g., height), males and females select their preferred walking speed in different ways: Females tend to have greater cadence on average and males tend to have larger SL. While some studies have found that height normalization controlled for sex differences in SL (22–25), others have found sex differences in SL to persist after controlling for body height (26–29). Our previous work found that females selected a greater cadence while maintaining SL normalized to body height at slightly faster, and much faster than normal walking speed conditions, suggesting that there may be sex-specific spatiotemporal strategies for increasing walking speed (30).

The first objective of this study was to characterize differences in spatiotemporal walking performance between different settings (laboratory treadmill, laboratory overground, hallway, indoor open, and outdoor pathway) during walking at different speeds for young adults. It was hypothesized that spatiotemporal walking performance would be different between treadmill and overground walking, and that parameters would differ between the laboratory settings and the non-laboratory settings with greater effects of settings outside of preferred walking speeds. The second objective of this study was to explore the interactions between sex and walking speed on spatiotemporal walking performance in different settings. Related to our second objective, it was hypothesized that the walking speed-related differences would depend on sex, with consistently greater cadence for females.

## 2 Methods

### 2.1 Participants

Young adults ages 18–35 years were recruited with advertisements through institutional channels and social media. Prospective participants were excluded if they self-reported a condition which impaired their walking balance control (e.g., current musculoskeletal injury and neurological impairment) and/or had not walked on a treadmill before. The study protocol received approval from the institutional research ethics board. All participants provided written informed consent prior to participation.

### 2.2 Settings

**Figure 1:**
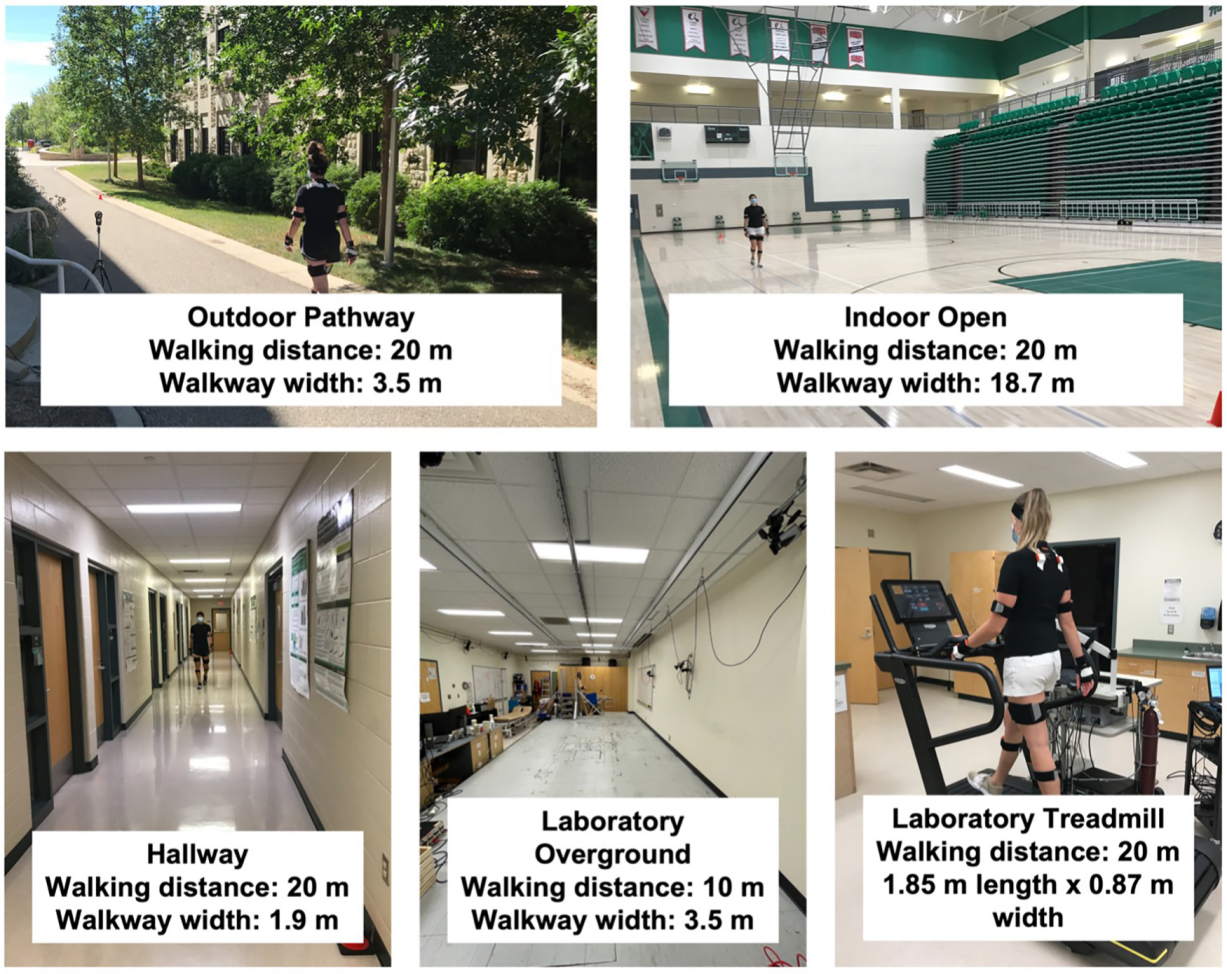
Data collection settings.

Participants walked in two laboratory settings [overground and treadmill (Skillrun Unity 7000, Technogym, Cesena, Italy)] and in three non-laboratory settings (a hallway, a large indoor open space, and an outdoor pathway) (Figure 1).

### 2.3 Protocol

In accordance with the sex and gender equity in research guidelines (31), data on participant sex and gender identity were collected by self-report. Prior to data collection, participants self-reported their sex assigned at birth (male, female, x) and their gender identity. The option “x” was provided for participants who were not assigned male or female sex at birth or chose not to disclose their sex. Gender identity was measured using a visual analog scale with man (0) and woman (100) on either end of the scale. Using a tablet-based visual analogue scale, participants were asked to slide the scale to match the gender identity that they align with.

A full body inertial sensor system (Xsens Awinda, Movella, Henderson, NV, fs = 60 Hz) was used to record kinematics. Participants were then equipped with 17 inertial sensors on the head, sternum, upper back, arms, wrists, hands, pelvis, thighs, legs, and feet using non-invasive straps, bands, and a shirt provided by the sensor system manufacturer.

The order of settings and walking speed conditions were block randomized for all participants with trials in each setting completed in randomized blocks and walking speed conditions in each setting completed in random order. In each setting, participants walked at different self-selected walking speeds following standardized verbal instruction: “much slower than normal” (SLOW); “comfortable or preferred” (PREF); and “much faster than normal” (FAST) (30). Participants were instructed to walk forward and to avoid shifting their attention away from the walking task. Pylons were placed 20 m apart at set locations in the non-laboratory settings to allow for defined start and stop points, while participants walked for 10 m across the entire walkway in the laboratory overground setting. Six trials of each speed were performed in each setting for a total of 90 trials. For the treadmill condition, participants completed a five-minute familiarization trial (32) during which they were asked to find their preferred walking speed while the treadmill display was covered. SLOW and FAST treadmills speeds were calculated based on previous experimental data (SLOW = 70% of the preferred speed, FAST = 130% of the preferred speed) (30). Participants walked at each calculated speed for a brief period and were asked if the calculated speed met the verbal description provided for each speed condition. Participants were permitted to adjust the treadmill speed if the calculated speed did not match their perception of the speed condition. Treadmill belt speeds were then fixed for the 20 m walking trials. The treadmill display of distance travelled was used to the define 20-meter treadmill walking trials.

Ambient temperature in all settings and wind speed in the outdoor setting were recorded at the time of data collection using a barometric anemometer (BT-100WM, Bestmeter Electronics Tecnhnology, Zhuhai Guangdong, China). Data collection was rescheduled when temperature was cooler than 5°C and/or when wind speed was greater than 40 km/hr and/or when there was rain or snowfall in the outdoor setting. Participants were rescheduled at temperatures cooler than 5°C to ensure sensor performance (33) and for participant comfort. As well, a qualitative description of the weather in the outdoor setting was recorded at the time of data collection. Since the non-laboratory settings were in public spaces, trials that included other people in the field of view of the participants were repeated. Excessive ambient noise that could impact participants' walking was not present in the public spaces.

### 2.4 Data analysis

Total body kinematic data were exported from the sensor system software (MVN Analyze 2019.2.1) and using custom routines (MATLAB 2019b, The MathWorks Inc., Natick, MA), the following outcome measures were then calculated: stride velocity, stride cadence, DS phase percentage, and SL normalized to participant body height (SL_N_). Stride velocity was calculated as stride length divided by stride time (m/s). Heel strike and toe off events were estimated using a manually tuned automated foot velocity threshold algorithm (34, 35). This algorithm has been validated for overground walking at slow (35) and preferred speeds (34, 35) and treadmill walking at preferred speeds (36, 37). Stride cadence was calculated as the inverse of the time between subsequent heel strikes of the right foot (i.e., stride time) multiplied by 60 (strides/min). DS phase percentage was calculated as the percentage of a stride with both feet in contact with the ground (%). SL_N_ was calculated as the anteroposterior distance between subsequent heel strikes of the right foot divided by body height [arbitrary units (au)]. We interpreted a decline in walking performance between settings to reflect the following differences in spatiotemporal walking parameters: decreased stride velocity (38, 39), decreased stride cadence (38, 39), decreased SL_N_ (38, 39), and increased DS phase percentage (38, 39). Data for the strides in the middle 5 m in the laboratory overground setting and strides in the middle 12 m in other settings were averaged and used for analysis.

### 2.5 Statistical analysis

Statistical analyses were conducted using IBM SPSS Statistics 28.0 (SPSS Inc., Armonk, NY). Data were checked for normality using Shapiro–Wilks tests. Independent samples *t*-tests were used to test for sex-based differences in mass, and body height. A Mann–Whitney *U*-test was used to test differences in median age between males and females as data were not normally distributed. For each speed condition, Wilcoxon signed rank tests were used to test for differences in the number of strides captured between settings since data were not normally distributed. To identify data outliers, an outlier routine using the generalized extreme Studentized deviate test was applied to remove stride and step outliers on a step-by-step or stride-by-stride basis for each variable (40). To prevent data distortion, a maximum of three strides or steps per trial were allowed to be declared an outlier. Sphericity was assessed using Mauchly's Test of Sphericity. Violations of sphericity were corrected using Greenhouse-Geisser epsilon. Adjusted degrees of freedom are provided for the outcomes which violated sphericity. A 5 × 3 × 2 (setting × walking speed condition × sex) repeated measures ANOVA was conducted for each spatiotemporal walking parameter. When interaction effects were present, simple main effects were investigated using repeated measures ANOVAs with Sidak pairwise comparisons. The pairwise comparisons from the simple main effects of setting are reported in the text and figures to help interpret significant setting interaction effects. For all statistical analyses, *p*-values <.05 were required for significant differences to be present. Effect size (*η_p_*^2^) and observed power are reported for the repeated measures ANOVAs.

## 3 Results

### 3.1 Demographics and setting information

Males were significantly taller than females, while there were no sex-based differences in age and mass (Table 1). Ambient temperatures for the settings included in this study are provided in Table 2. Outdoor weather conditions were sunny (*n *= 10), partly cloudy (*n* = 11), and cloudy (*n* = 21). The number of steps and strides averaged for each participant ranged from 816 to 1,612 steps and 408–806 strides (Table 3 for strides in each walking condition). For each speed condition, there were significant differences (*p* < .05) in the average number of strides captured in each setting (laboratory treadmill > hallway > indoor open > outdoor pathway > laboratory overground) (Table 3).

**Table 1:** Demographics of study participants.

	Males (*n* = 21)	Females (*n* = 21)	*p*	Total (*n* = 42)
Gender VAS (cm)^a^	0 (0–7)	100 (75–100)		
Age (yrs)	23 ± 4	24 ± 5	.757^b^	24 ± 5
Mass (kg)	78 ± 15	69 ± 16	.073	74 ± 17
Body height (cm)	179 ± 8	170 ± 7	<.001	174 ± 9

Data are presented as mean ± standard deviation unless otherwise indicated.

^a^ Mode (range) of data are provided.

^b^ A Mann–Whitney *U*-test was used to test for sex-based differences in age distribution.

**Table 2:** Ambient temperature and wind speed.

	Laboratory treadmill	Laboratory overground	Hallway	Indoor open	Outdoor pathway
Ambient temperature (°C)	21.4 ± .8 (19.6–23.0)	22.7 ± .6 (21.4–23.8)	22.8 ± 0.6 (21.3–23.7)	22.3 ± 0.8 (19.7–24.3)	19.8 ± 5.9 (10.9–29.1)
Wind speed (M/S)					1.6 ± 0.6 (0–2.7)

Data are presented as mean ± standard deviation (range).

**Table 3:** Mean ± standard deviation (range) number of strides captured for each walking condition across participants.

	Laboratory treadmill	Laboratory overground	Hallway	Indoor open	Outdoor pathway
SLOW	63 ± 10^A^ (47–94)	17 ± 5^B^ (9–32)	53 ± 10^C^ (35–83)	51 ± 9^D^ (35–75)	48 ± 9^E^ (32–68)
PREF	51 ± 10^A^ (34–79)	11 ± 3^B^ (6–22)	41 ± 7^C^ (29–63)	40 ± 6^D^ (28–58)	38 ± 6^E^ (27–56)
Fast	40 ± 8^A^ (29–64)	7 ± 2^B^ (6–15)	32 ± 4^C^ (24–41)	31 ± 4^D^ (24–39)	30 ± 4^E^ (23–37)

At each speed condition, Wilcoxon signed rank tests were used to test for setting differences.

At each speed condition, settings with different superscript letters (A, B, C, D, E) were significantly different at *p* < .05.

### 3.2 Spatiotemporal walking parameters

The mean ± standard deviation of the included spatiotemporal walking parameters for the walking conditions are provided disaggregated by sex and for the total sample (Table 4).

**Table 4:** Mean ± standard deviation of the included spatiotemporal walking parameters for the walking conditions.

Stride velocity (m/s)				
Speed condition	Setting	Male (*n* = 21)	Female (*n* = 21)	Total (*n* = 42)
SLOW	Laboratory Treadmill	.68 ± .2	.68 ± .1	.68 ± .1
	Laboratory Overground	.81 ± .2	.80 ± .2	.81 ± .2
	Hallway	.87±.2	.84 ± .2	.86 ± .2
	Indoor Open	.91 ± .2	.89 ± .2	.90 ± .2
	Outdoor Pathway	.99 ± .2	1.00 ± .2	.99 ± .2
PREF	Laboratory Treadmill	1.02 ± .2	.98 ± .2	1.00 ± .2
	Laboratory Overground	1.31 ± .2	1.29 ± .2	1.30 ± .2
	Hallway	1.32 ± .2	1.32 ± .2	1.32 ± .2
	Indoor Open	1.38 ± .2	1.38 ± .2	1.38 ± .2
	Outdoor Pathway	1.46 ± .2	1.48 ± .2	1.47 ± .2
FAST	Laboratory Treadmill	1.46 ± .3	1.39 ± .2	1.42 ± .3
	Laboratory Overground	1.91 ± .2	1.82 ± .2	1.87 ± .2
	Hallway	1.94 ± .2	1.81±.2	1.88 ± .2
	Indoor Open	1.99 ± .2	1.93 ± .2	1.96 ± .2
	Outdoor Pathway	2.05 ± .2	1.97 ± .2	2.01 ± .2
Stride cadence (strides/min)				
Speed condition	Setting	Male (*n* = 21)	Female (*n* = 21)	Total (*n* = 42)
SLOW	Laboratory Treadmill	39 ± 6	42 ± 4	40 ± 5
	Laboratory Overground	42 ± 5	42 ± 6	42 ± 5
	Hallway	43 ± 5	44 ± 6	43 ± 5
	Indoor Open	44 ± 5	45 ± 6	44 ± 5
	Outdoor Pathway	46 ± 5	47 ± 5	46 ± 5
PREF	Laboratory Treadmill	48 ± 5	51 ± 5	50 ± 5
	Laboratory Overground	52 ± 3	55 ± 3	54 ± 4
	Hallway	53 ± 3	56 ± 3	54 ± 3
	Indoor Open	54 ± 4	57 ± 3	55 ± 4
	Outdoor Pathway	55 ± 3	58 ± 3	57 ± 4
FAST	Laboratory Treadmill	56 ± 5	59 ± 5	58 ± 5
	Laboratory Overground	62 ± 5	66 ± 5	64 ± 5
	Hallway	62 ± 5	66 ± 5	64 ± 5
	Indoor Open	64 ± 5	68 ± 5	66 ± 5
	Outdoor Pathway	65 ± 5	68 ± 5	66 ± 5
DS phase percentage (%)				
Speed condition	Setting	Male (*n* = 21)	Female (*n* = 21)	Total (*n* = 42)
SLOW	Laboratory Treadmill	33 ± 4	34 ± 4	34 ± 4
	Laboratory Overground	31 ± 5	31 ± 5	31 ± 5
	Hallway	30 ± 5	31 ± 4	30 ± 4
	Indoor Open	29 ± 5	29 ± 3	29 ± 4
	Outdoor Pathway	27 ± 4	27 ± 3	27 ± 4
PREF	Laboratory Treadmill	29 ± 4	30 ± 4	29 ± 4
	Laboratory Overground	24 ± 4	24 ± 3	24 ± 3
	Hallway	24 ± 4	24 ± 3	24 ± 3
	Indoor Open	23 ± 4	23 ± 3	23 ± 3
	Outdoor Pathway	22 ± 4	22 ± 3	22 ± 3
FAST	Laboratory Treadmill	24 ± 5	24 ± 3	24 ± 4
	Laboratory Overground	18 ± 3	19 ± 2	18 ± 3
	Hallway	18 ± 3	19 ± 2	18 ± 2
	Indoor Open	17 ± 3	18 ± 2	18 ± 2
	Outdoor Pathway	17 ± 3	18 ± 2	17 ± 2
SL_N_ (au)				
Speed condition	Setting	Male (*n* = 21)	Female (*n* = 21)	Total (*n* = 42)
SLOW	Laboratory Treadmill	.58 ± .08	.57 ± .07	.58 ± .08
	Laboratory Overground	.64 ± .10	.66 ± .08	.65 ± .09
	Hallway	.67 ± .11	.68 ± .07	.67 ± .10
	Indoor Open	.69 ± .10	.70 ± .07	.69 ± .09
	Outdoor Pathway	.73 ± .11	.74 ± .07	.73 ± .09
PREF	Laboratory Treadmill	.70 ± .11	.68±.09	.69 ± .10
	Laboratory Overground	.83 ± .10	.82 ± .07	.83 ± .8
	Hallway	.83 ± .10	.83 ± .07	.83 ± .09
	Indoor Open	.86 ± .10	.86 ± .07	.86 ± .08
	Outdoor Pathway	.89 ± .11	.90 ± .07	.90 ± .09
FAST	Laboratory Treadmill	.86 ± .13	.83 ± .09	.84 ± .11
	Laboratory Overground	1.03 ± .09	.98 ± .07	1.00 ± .08
	Hallway	1.03 ± .09	.98 ± .06	1.00 ± .08
	Indoor Open	1.04 ± .07	1.00 ± .06	1.02 ± .07
	Outdoor Pathway	1.06 ± .08	1.03 ± .06	1.04 ± .08

#### 3.2.1 Stride velocity

**Figure 2:**
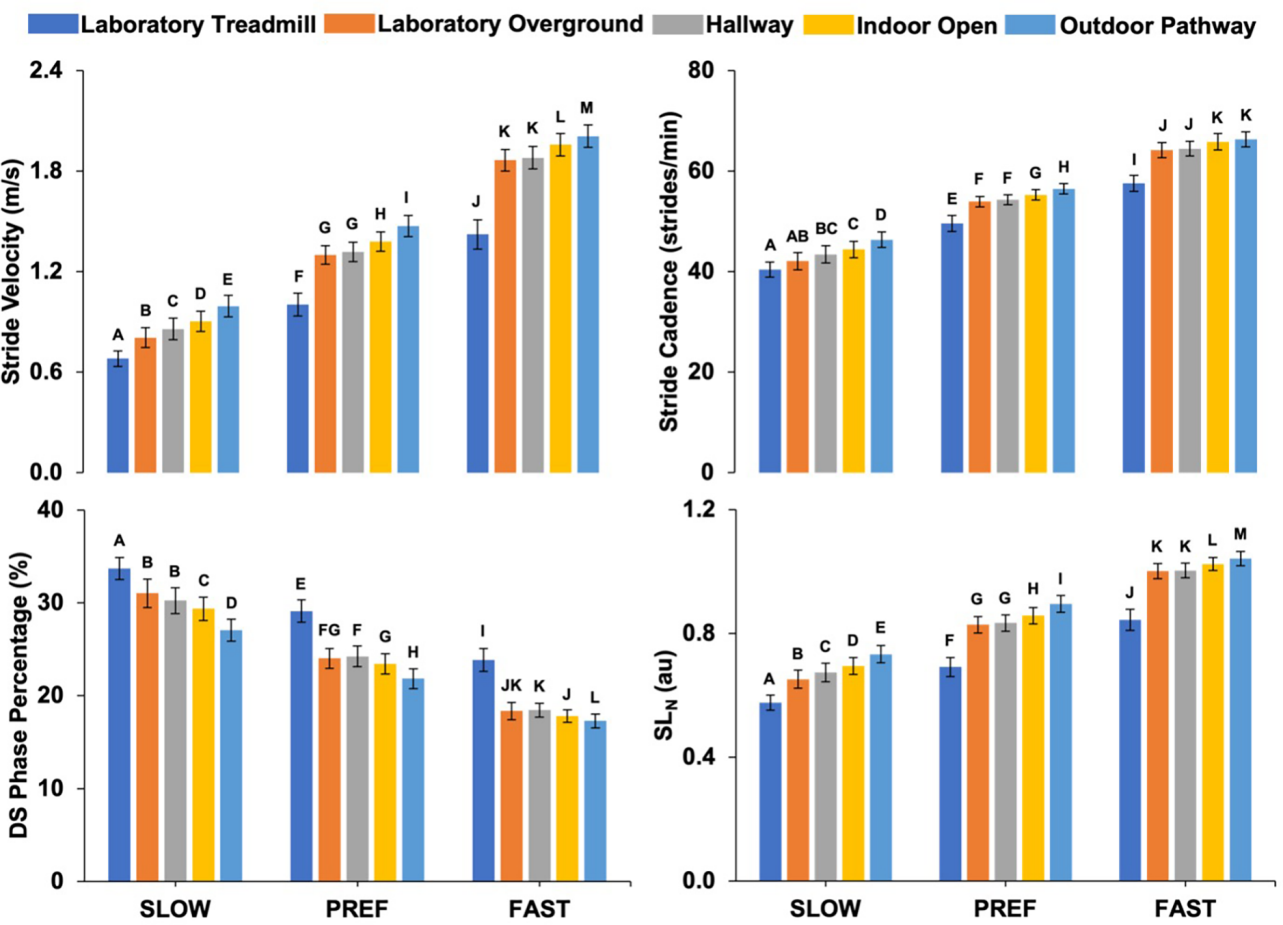
Setting × walking speed condition interaction effects for spatiotemporal walking parameters (mean ± 95% confidence interval). For each walking speed condition and spatiotemporal walking parameter, settings with different letters above their error bars are significantly different at *p* < .05.

There was a setting × walking speed condition interaction effect for stride velocity [*F* (2.512, 100.481) = 25.565, *p* < .001, *η_p_*^2^ = .390, observed power = 1.000] (Figure 2). At the SLOW walking speed, stride velocity was different between all settings (laboratory treadmill < laboratory overground < hallway < indoor open < outdoor pathway). At PREF and FAST walking speeds, stride velocity was lower in the laboratory treadmill setting compared to the other settings; lower in the laboratory overground and hallways settings compared to the indoor open and outdoor pathway settings; and lower in the indoor open setting compared to the outdoor pathway setting.

#### 3.2.2 Stride cadence

There was a setting × walking speed condition interaction effect for stride cadence [*F* (2.807, 112.273) = 10.159, *p* < .001, *η_p_*^2^ = .203, observed power = .997] (Figure 2). At the SLOW walking speed, stride cadence was lower in the laboratory treadmill setting compared to the hallway, indoor open, and outdoor pathway settings; lower in the laboratory overground setting compared to the indoor open and outdoor pathway settings; lower in the hallway setting compared to the outdoor pathway setting; and lower in the indoor open setting compared to the outdoor pathway setting. At the PREF walking speed, stride cadence was lower in the laboratory treadmill setting compared to the other settings; lower in the laboratory overground and hallway settings compared to the indoor open and outdoor pathway settings; and lower in the indoor open setting compared to the outdoor pathway setting. At the FAST walking speed, stride cadence was lower in the laboratory treadmill setting compared to the other settings; and lower in the laboratory overground and hallway settings compared to the indoor open and outdoor pathway settings. There was a significant main effect of sex for stride cadence [*F* (1, 40) = 5.871, *p *= .020, *η_p_*^2^ = .128, observed power = .657] with greater stride cadence (mean ± standard deviation) for females (55 ± 4.6 strides/min) compared to males (53 ± 4.6 strides/min).

#### 3.2.3 DS phase percentage

There was a setting × walking speed condition interaction effect for DS phase percentage [*F* (2.896, 115.851) = 9.667, *p* < .001, *η_p_*^2^ = .195, observed power = .996] (Figure 2). At the SLOW walking speed, DS phase percentage was greater in the laboratory treadmill setting compared to the other settings; greater in the laboratory overground and hallways setting compared to the indoor open and outdoor pathway settings; and greater in the indoor open setting compared to the outdoor pathway setting. At PREF and FAST walking speeds, DS phase percentage was greater in the treadmill setting compared to the other settings; greater in the laboratory overground setting compared to the outdoor pathway setting; greater in the hallway setting compared to the indoor open and outdoor pathway settings; and greater in the indoor open setting compared to the outdoor pathway setting.

#### 3.2.4 Normalized stride length (SLN)

There was a setting × walking speed condition interaction effect for SL_N_ [*F* (2.735, 109.402) = 12.053, *p* < .001, *η_p_*^2^ = .232, observed power = .999] (Figure 2). At the SLOW walking speed, SL_N_ was different between all settings (laboratory treadmill < overground < hallway < indoor open < outdoor pathway). At the PREF and FAST walking speeds, SL_N_ was smaller in the laboratory treadmill setting compared to the other settings; smaller in the laboratory overground and hallway settings compared to the indoor open and outdoor pathway settings; and smaller in the indoor open setting compared to the outdoor pathway setting. A walking speed × sex interaction effect was present for SL_N_ [*F* (1.495, 59.781) = 5.289, *p* = .014, *η_p_*^2^ = .117, observed power = .735]. The pairwise comparisons indicated there were no statistical differences in SL_N_ between male and female groups at each walking speed condition.

## 4 Discussion

The current study characterized differences in spatiotemporal walking performance between different settings during walking at different speeds for young adults. Spatiotemporal walking performance differences between settings were not the same for the different walking speed conditions. For real-world walking assessments, our research suggests that walking assessments should be conducted in the same or similar settings due to the potential impact of setting characteristics and that walking performance may differ slightly between different indoor settings (e.g., a hallway vs. an open space) and between indoor and outdoor settings. Sex differences in spatiotemporal walking performance were independent of setting and walking speed conditions for cadence and depended on walking speed condition for SL_N_. Our work suggests that sex is an important factor to consider for accurate walking data interpretations in and across different settings.

### 4.1 Effect of settings

Stride velocity differed significantly between settings. Since the walking distance assessed in the analysis was equal across the longer 20-meter settings (i.e., laboratory treadmill, hallway, indoor open, and outdoor pathway), the observed differences in the number of strides captured across these settings is likely driven by the observed differences in stride velocity between settings and the consistency of the number of strides captured within each participant. At PREF and FAST, differences in stride velocity between the laboratory treadmill setting and overground settings were greater than the clinically meaningful threshold of 0.2 m/s (1). Although differences in stride velocity between overground settings were below the clinically meaningful threshold, setting influences on stride velocity could impact the interpretation of health risk assessments if other populations differ their stride velocity in a similar manner between settings (1). Recent studies have suggested that settings influence spatiotemporal walking performance in a similar manner for older adults (8, 17) and that the effects of settings appear to be greater than the effects of age on walking performance (8, 17). These are important findings to consider since a reduced walking speed when tested in a hallway compared to in an open space may impact health risk status assessments (1). The observed differences in stride velocity between settings are important to consider for health risk assessments, especially with the increased use of wearable sensors for gait assessment outside of lab settings (2).

Setting-related influences on stride velocity found in this study align with prior research in young adults which found slower self-selected treadmill speeds when compared to self-selected overground speeds (3–6, 8) and faster walking speeds outdoors when compared to indoors (8, 11, 12, 15). Our work extends previous research by demonstrating that stride velocity is influenced by different indoor settings. Setting-related differences in stride velocity were likely due to differences in stride cadence and SL_N_ between settings (20, 41). Stride velocity differences between settings may have also led to differences in DS phase percentage (19).

### 4.2 Effect of settings at different walking speeds

Spatiotemporal walking performance differences between settings depended on the walking speed condition. Fewer statistical differences between settings were detected for stride cadence and DS phase percentage outside of preferred walking speeds. The lack of detectable differences could be partly related to increased variability in stride cadence and DS phase percentage outcomes at SLOW speeds (18), though further investigation is required. In contrast, the pattern of differences between settings at the FAST speeds was different than at SLOW and PREF speeds providing some evidence that the speed by setting interaction was not solely due to variability. When stride cadence was similar between settings at SLOW and FAST speeds, differences in stride velocity and SL_N_ were present suggesting that differences in chosen stride velocity between these settings may have been driven by setting differences in SL_N_ and not stride cadence. These results suggest that settings may have a greater influence on spatial aspects of performance when compared to temporal aspects of performance when control requirements are influenced.

### 4.3 Potential effects of setting characteristics

Characteristics of the different settings may have led to differences in spatiotemporal walking performance. The presence of lateral pathway boundaries defining the walkway may influence walking performance by providing visual cues for the online control of walking (42). For example, differences in walking performance were almost always present between the hallway (where the walls restricted participant movement in the lateral directions) and indoor open setting (where there were no nearby walkway boundaries regulating behavior). Due to the different characteristics in the settings included in our study, we are unable to ascertain the independent effects of walkway boundary definition on walking for young adults. Therefore, future work should clarify the effects of walkway definition on spatiotemporal walking performance to better understand the setting differences observed in the current study.

Since individuals with balance challenges (e.g., older adults) often rely more on vision (43), their walking could be influenced by visual cues provided in different settings (42) to a greater extent when compared to younger adults. Visual perception of the environment may only partially explain differences in walking behavior between different settings; however, as prior work has suggested that differences in walking between indoor and outdoor settings persist during blindfolded walking (12, 13). Thus, an understanding of the factors in settings which influence walking based on, and independent of visual perception is required.

### 4.4 Effects of sex

Our research further demonstrates evidence of sex-based differences in spatiotemporal walking performance. In alignment with prior work, females had greater cadence, while SL_N_ was not different between males and females (21–25). These differences are in contrast to some previous research reporting sex differences in SL after controlling for height (26–29), which may be due to differing protocols and sample populations indicating the need for further investigation. Absence of sex-based differences in stride velocity supports the idea that females may select a greater cadence to establish their self-selected walking speeds (30). The interaction between sex and walking speed conditions for SL_N_ provides further evidence of sex-specific strategies to increase walking speed (30).

### 4.5 Limitations

The shorter walkway in the lab overground setting led to fewer strides captured when compared to other settings. Consequently, the comparisons between the lab overground setting and the other settings may be confounded by differences in the number of strides captured since natural gait variations may have a greater influence when fewer strides are used to calculate means (44, 45). The method used to establish preferred treadmill walking speeds is unique to this study protocol and the selected treadmill walking speeds could vary from selected overground speeds if a different protocol was used. Additionally, results may not generalize to self-paced treadmills and treadmills with different dimensions. Data for walking outcomes may not generalize to walking outside of the scope of the current study due to potential carry-over effects between the walking speed conditions. It should be noted that the validity and sensitivity of the footfall detection algorithm used is fairly well established for a variety of conditions but has not been specifically tested in fast overground walking speeds or for outdoor settings and may have larger event detection offsets from direct measurement at slower walking speeds (34). Data were collected during the Covid-19 pandemic and participants may have felt anxious during data collection, particularly in the non-laboratory settings where there was potential for participants to come into close contact with other people. Additionally, as we did not collect data with other people present in these settings, we are not certain if walking performance would be the same with other people present (e.g., pedestrians walking along the outdoor pathway). We considered conducting both a sex- and gender-based analysis for this project, but since all participants in our study identified as cis-gender, we proceeded with a sex-based analysis only. As we all participants in this study self-reported as cis gender, the extent to which results may be attributed to participant sex vs. gender is not known. The impact of settings on the variability of walking outcomes was not examined in this study and should be examined in future research.

## 5 Conclusion

Spatiotemporal walking performance was affected by settings, particularly at slow walking speeds. The characteristics of experimental set-ups such as walkway definition and length, and participant sex are important factors to consider when designing research studies and comparing the results of different research studies. In addition, health practitioners should consider setting-based differences in spatiotemporal parameters when creating and evaluating rehabilitation strategies.

## Data Availability

The raw data supporting the conclusions of this article will be made available by the authors, without undue reservation.
